# Dynamic screening strategies for the control of a prolonged vancomycin-resistant enterococci outbreak: A decade-long study in a Japanese tertiary hospital

**DOI:** 10.1017/S0950268826101344

**Published:** 2026-04-07

**Authors:** Hiroyuki Shimizu, Tomoko Kawada, Tomoyuki Osumi, Masashi Sakai, Noboru Yoshimoto, Atsuo Sato, Masanori Nishikawa

**Affiliations:** 1 Department of Clinical Laboratory Medicine, Fujisawa City Hospital, Japan; 2 Department of Clinical Laboratory, Fujisawa City Hospital, Japan; 3 Department of Pharmacy, Fujisawa City Hospital, Japan; 4 Department of Nephrology, Fujisawa City Hospital, Japan; 5 Department of Respiratory Surgery, Fujisawa City Hospital, Japan; 6 Department of Emergency Pediatrics, Fujisawa City Hospital, Japan; 7 Department of Respiratory Medicine, Fujisawa City Hospital, Japan

**Keywords:** case–control study, nosocomial, outbreak, screening, vancomycin-resistant enterococci

## Abstract

In low-prevalence settings, the epidemiological yield of screening strategies for controlling vancomycin-resistant enterococci (VRE) outbreaks has not been fully established. We retrospectively analysed a prolonged VRE outbreak at a 536-bed tertiary-care hospital in Japan from 2010 to 2021 to evaluate sequential screening strategies across epidemic phases and to identify risk factors for VRE acquisition. Hospital-wide, admission-based, antimicrobial exposure-based, passive, and haemodialysis-targeted screening strategies were implemented over time. Screening yields were compared longitudinally, and a retrospective case–control study was performed using data from the initial hospital-wide screening phase. Molecular epidemiology was assessed by pulsed-field gel electrophoresis (PFGE). In total, 169 VRE-positive patients were identified, including seven infections and 162 asymptomatic carriers. Hospital-wide screening in the early epidemic phase showed the highest positivity rate (0.91%), whereas targeted strategies consistently yielded lower rates (0.09–0.34%). Haemodialysis, specific oral care practices, and prior exposure to carbapenems, glycopeptides, and piperacillin/tazobactam were independently associated with VRE acquisition. PFGE revealed substantial genetic diversity, suggesting sustained nosocomial transmission with repeated introductions. Early broad-based screening may be epidemiologically efficient in the initial phase of VRE outbreaks in low-prevalence settings, followed by adaptive refinement for long-term control.

## Introduction

Vancomycin-resistant enterococci (VRE) is a generic term for enterococci that have acquired resistance to vancomycin. The first reported case of VRE was in the United Kingdom in 1988 [1]. VRE causes various healthcare-associated infections, including urinary tract infections, cholangitis, cholecystitis, and infective endocarditis, despite their relatively low virulence [2]. Around 10% of colonized patients develop a clinical infection within 30 days [3]. VRE bacteraemia increases mortality 1.8–2.5 times compared to vancomycin-susceptible strains [4, 5]. Outbreaks of VRE are often protracted and require intensive, multifaceted infection control interventions, resulting in a substantial economic burden [6]. A Canadian study reported that VRE infections incurred an average additional cost of $17949 and prolonged hospital stays by 13.8 days per patient [7].

Given these important clinical and economic consequences, preventing nosocomial transmission of VRE is of paramount importance. Effective control strategies require identification of patients with active VRE infection and those who are colonized with VRE. Screening and isolating VRE carriers is a cost-effective infection prevention and control intervention [8–11]; however, the optimal timing, target population, and duration of VRE screening remain unclear. This study aimed to evaluate the effectiveness of multiple screening strategies for VRE outbreak control and to identify risk factors for VRE acquisition during the initial hospital-wide screening.

## Methods

### Study setting and periods

In this retrospective study, we analysed the overall course of a VRE outbreak and subsequent ongoing screening tests conducted at Fujisawa City Hospital, a 536-bed tertiary care centre in Japan, between May 2010 and March 2021. The hospital features six dedicated infectious disease beds, with average patient length of stay of 13.2 days.

### Infection control measures

To implement VRE infection control measures, we referenced several guidelines developed in other countries [12–14], which include antimicrobial stewardship, screening tests, patient isolation, environmental cleaning, and adherence to contact infection control measures. Based on these references, we implemented various measures to prevent the spread of VRE (Table 1). These measures aimed to prevent an increase in the number of infected patients, reduce the bacterial burden via antimicrobial stewardship, and promote early detection of VRE colonization or infection using various screening tests. We conducted a case – control study to analyse the risk factors approximately 7 months after we identified the first case. We primarily followed five screening strategies: hospital-wide, admission, antimicrobial, passive, and haemodialysis screening. Admission screening was performed for all patients upon admission. Antimicrobial screening was performed among patients who had received glycopeptides, third- and fourth-generation cephalosporins, beta-lactamase inhibitor combinations, carbapenems, aminoglycosides, or clindamycin for ≥7 days [15, 16]. Passive screening is an automated screening method to detect VRE when routine stool culture is submitted to identify patients not captured in antimicrobial screening. Haemodialysis screening was performed every 2 weeks in patients undergoing haemodialysis during hospitalization. In addition to the five primary screening tests, we performed routine screening of wards with VRE-positive patients and screening upon transfer of these patients to other wards. Furthermore, to promptly and effectively control the outbreak, we organized meetings to discuss countermeasures that included external experts in microbiology, infection control, infectious disease treatment, medical epidemiology, and public health.

**Table 1.** Measures to control the spread of VRE infection

Measures to prevent the increase of infected patients	・ Ensure and evaluate medical staff compliance with infection prevention measures.
	・ Cohort management of infected patients in dedicated wards.
	・ Temporary private isolation of patients with suspected VRE infection.
	・ Reinforcement and thoroughness of environmental cleaning.
	・ Hand hygiene instruction for patients and visitors.
Measures to prevent an increase in the amount of bacteria in the infected patient’s body	・ Reinforce appropriate use of antimicrobial agents.
	・ Recommendation of oral administration of BIO-THREE® (containing *Clostridium butyricum*, *Enterococcus faecium*, and *Bacillus subtilis*) to VRE-infected patients.
Early detection of patients infected with VRE	・ Comprehensive screening of all hospitalized patients.
	Hospital-wide screening
	Admissions screening
	・ Limited screening of high-risk inpatients.
	Antimicrobial screening
	Passive screening
	Haemodialysis screening
Other measures	・ Providing information and awareness to medical staff.
	・ Risk factor analysis through epidemiological studies.
	・ Outpatient follow-up of VRE-infected patients.
	・ Regional collaboration with other hospitals and facilities.

Abbreviation: VRE, vancomycin-resistant enterococci.

### Microbiologic testing

Rectal swabs were collected from patients by nurses using BD BBL CultureSwab Plus™ (Nippon Becton Dickinson Co. Ltd., Tokyo, Japan) for VRE screening. The swabs were directly inoculated onto BD VRE Selective Agar (Nippon Becton Dickinson Co. Ltd., Tokyo, Japan). The agar plates were divided into two sections. A seed swab was directly applied to one section and streaked using a 1-μL inoculating loop. The agar plates were incubated aerobically at 35 C for 48 h, with colony observation performed every 24 h. If suspected colonies were observed, Gram staining was performed. Gram-positive cocci in short chains were considered presumptive VRE and were sub-cultured onto blood agar for pure culture isolation. Colonies grown in pure culture were subjected to catalase and PYR tests (SwabColor PYR; Iwaki & Co., Ltd., Tokyo, Japan). Isolates exhibiting a negative catalase test and a positive PYR test were presumptively identified as *Enterococcus* species. Species identification and antimicrobial susceptibility testing were then performed using the MicroScan WalkAway 40 system (Siemens Healthineers, Erlangen, Germany) with the Prompt method for bacterial suspension preparation. The MicroScan WalkAway Pos Combo 3.1 J Panel was used to determine minimum inhibitory concentration values. VRE was confirmed if the minimum inhibitory concentration for vancomycin was >16 μg/mL for *Enterococcus* species.

Detection of vancomycin resistance genes was performed using primers described by Dutka-Malen et al. [17].

### Molecular typing of VRE isolates

To investigate the genetic relatedness among the VRE isolates, molecular typing was performed using pulsed-field gel electrophoresis (PFGE). Agarose gel plugs containing bacterial cells were prepared and digested with the restriction enzyme *Sma*I (BioLabs, Ipswich, MA, USA). Electrophoresis was conducted using the CHEF DRII system (Bio-Rad Laboratories, Hercules, CA, USA) under the following conditions: 6.0 V/cm at 14 C. Phylogenetic trees were constructed based on the banding patterns using Fingerprinting II software (Bio-Rad Laboratories). Isolates showing PFGE patterns with a difference of up to three-fragment difference were defined as the same clone [18].

### Case–control study

To identify risk factors associated with VRE acquisition, a retrospective case–control study was conducted using VRE-positive cases identified during the initial hospital-wide screening conducted in December 2010 (*n* = 23). We retrospectively collected patient data from electronic medical records, including age, sex, pre-admission residence, activities of daily living, presence or absence of enteral feeding, dependency on assistance with hygiene and toileting, presence of an indwelling urinary catheter, diaper use, oral care practices, airway suctioning requirements, history of intensive care unit admission, medical procedures performed, use of medical devices, and prior exposure to antibiotics. Age was analysed as a continuous variable. Prior antibiotic exposure was treated as a categorical variable, defined as exposure to specific antimicrobial agents within the preceding 3 months. Sensitivity analyses were not performed because all eligible cases and controls from the initial hospital-wide screening were included, leaving no scope for alternative analytic scenarios.

This retrospective case–control cohort was defined by the initial hospital-wide screening at the start of the outbreak. Patients for whom consent was not obtained were excluded. We included every VRE-positive patient detected at that screening (*n* = 23) and all other inpatients across all wards who underwent the same screening round and tested negative for VRE (*n* = 376). All inpatients were screened during this round, and no patients had missing core clinical data. Because the cohort was fixed by this screening round rather than by a target sample size, no a priori sample size calculation was performed. This approach ensured that all available cases were included, thereby maximizing statistical power and minimizing selection bias.

### Statistical analysis

All statistical analyses were conducted using IBM SPSS Statistics 29.0 software (IBM Corp., Armonk, NY, USA). Risk factor analysis was performed using the chi-square test, and a *p*-value of <0.05 was considered statistically significant. In the case–control study, univariate analysis was performed to statistically assess various potential risk factors hypothesized associated with VRE acquisition. Multivariable logistic regression was not performed due to the limited number of cases (*n* = 23).

### Ethical considerations

Management and infection control interventions for outbreaks were conducted as part of normal infection control programs. This study was approved by the ethical review committee of Fujisawa City Hospital (approval number F2023003). To ensure participant autonomy, an opt-out option was provided.

## Results

### Outbreak overview

Table 2 summarizes the timeline of implemented infection control measures and important events. In May 2010, VRE was detected in the bile of a 72-year-old male patient who was hospitalized with acute cholangitis, marking the index case in this outbreak. Standard precautions, including hand hygiene, were promptly reinforced, and thorough environmental cleaning was implemented. Specifically, to interrupt indirect contact transmission via contaminated environmental surfaces, we focused on disinfecting frequently touched surfaces, such as shared toilets, bed rails, bedside tables, and handrails. To ensure the quality of cleaning, a head nurse with infection control nurse certification conducted checks on cleaning procedures and provided feedback to cleaning staff and/or contractors. Simultaneously, screening of all patients in the same ward revealed VRE carriage in an additional 13 patients, all of whom were promptly placed in single rooms or cohort isolation. Given the continued increase in VRE-positive cases, a task force meeting with external experts was convened in December 2010. Following this meeting, multiple screening tests were sequentially introduced. By October 2011, all VRE-positive inpatients were discharged.

**Table 2.** Overview of events related to the VRE outbreak and the implemented infection control measures and the number of VRE positive patients

Phase	Year	Month	Events	number of symptomatic patients	number of asymptomatic carriers
Phase 1	2010	May	Recognition of first case of VRE infection		
	2010	Dec	Initial task force meeting with outside experts		
			Initial hospital-wide screening		
			Initiate admission screening		
	2011	Feb	Initiate antimicrobial screening		
			Initiate passive screening		
			Initiate screening of haemodialysis patients	6	100
Phase 2	2011	Oct	All VRE-positive inpatients were discharged	0	23
Phase 3	2013	Sep	Admission screening was reduced from all patients to high-risk patients		
	2014	Mar	Discontinue screening of haemodialysis patients	1	39
Phase 4	2018	Jan	Discontinue hospital-wide screening (No new detection for more than 9 months since the last detection)		
	2018	Aug	Discontinue passive screening		
	2021	Mar	Discontinue admission screening		
			Discontinue antimicrobial screening	0	0

Abbreviation: VRE, vancomycin-resistant enterococci.

**Figure 1.**
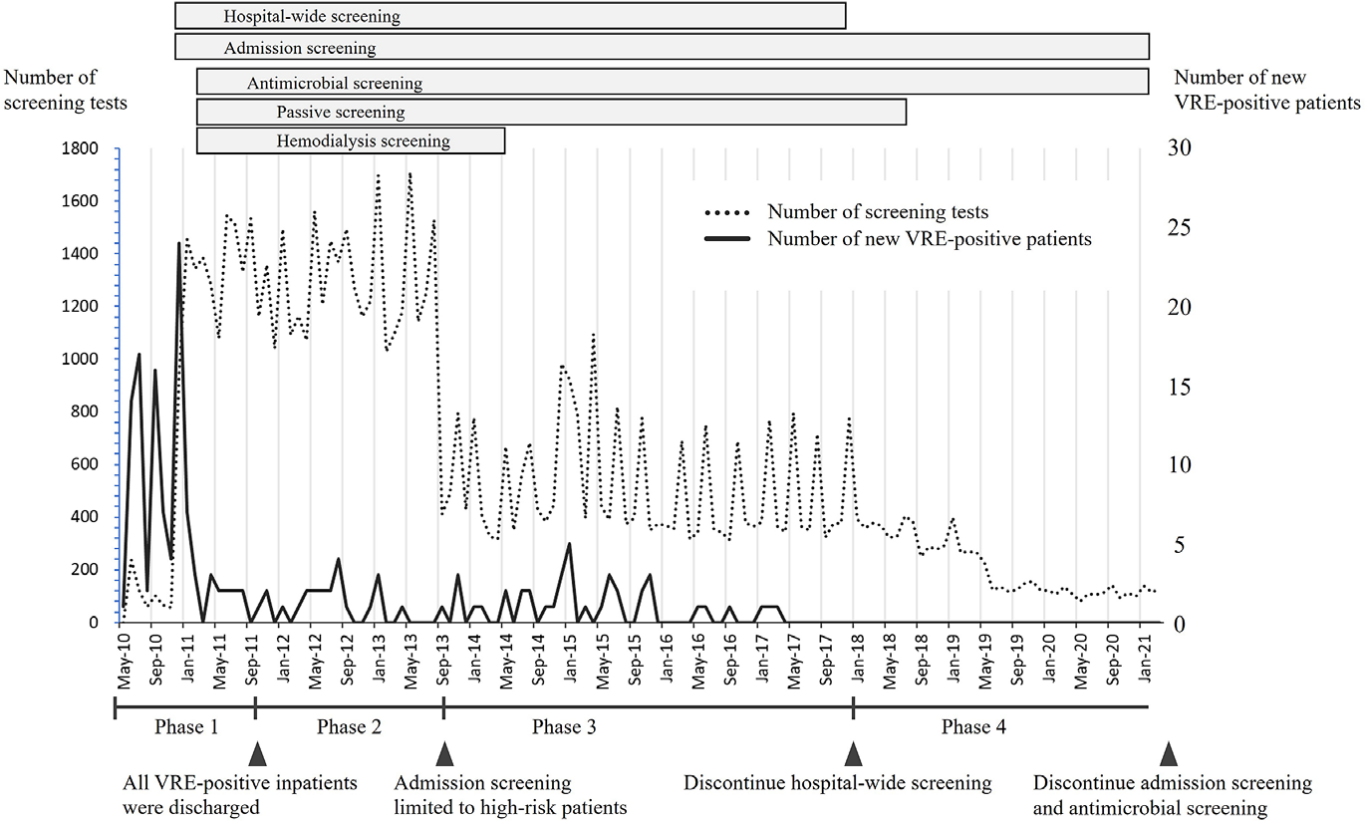
Epidemiologic curve showing the number of patients with newly detected vancomycin-resistant enterococci (VRE; solid line) and number of screening tests performed (dashed line). The transient sharp increases observed in the number of screenings correspond to the periodic implementation of hospital-wide screening strategies. No additional VRE cases were detected after March 2017, and hospital-wide screening was discontinued in January 2018 following a 9-month period with no newly identified cases.

Over the approximate 10-year period from May 2010 to March 2021, a total of 169 cases of VRE were detected, comprising seven symptomatic patients and 162 asymptomatic carriers identified through screening. Figure 1 displays the epidemiologic curve alongside screening tests performed. The study period was divided into four distinct phases: May 2010–October 2011 (phase 1), October 2011–September 2013 (phase 2), September 2013–December 2017 (phase 3), and January 2018–March 2021 (phase 4). Phase 1 was defined as the period from initial outbreak recognition to the point when all VRE-positive inpatients were discharged. Phase 2 was defined as the period during which efforts were made to identify potential VRE carriers by continuing various screening procedures initiated in phase 1. Phase 3 was defined as the period when admission screening was limited to high-risk patients. High-risk patients were defined as those with a history of VRE detection, diaper use, inter-facility transfer, or pre-admission antibiotic use for ≥7 days. Phase 4 was defined as the period between the reduction in hospital-wide screening owing to two consecutive instances with no newly detected cases, and March 2021 when all VRE screening tests were discontinued owing to no positive results in the remaining admission, antimicrobial, and passive screening.

### Risk factor analysis

Table 3 presents the results of a case–control study conducted during the initial hospital-wide screening (VRE positive *n* = 23, VRE negative *n* = 376). Oral care practices, haemodialysis, and prior exposure to carbapenems, glycopeptides, and piperacillin/tazobactam were identified as significant risk factors for VRE acquisition.

**Table 3.** Comparison of risk factors between patients with VRE and a control group

	VRE-positive patient (*n* = 23)	VRE-negative patient (*n* = 376)	RR	95% CI	*p* value
Age	68.6 ± 15	64.9 ± 17.5			
Male gender	15 (65.2%)	214 (56.9%)	1.39	0.60–3.21	0.572
Place of residence prior to hospitalization					
Home	20 (87.0%)	359 (95.5%)	2.84	0.92–8.77	0.185
Facility	3 (13.0%)	17 (4.5%)			
Activities of daily living (ADL)					
Independent	5 (21.7%)	118 (31.4%)			
Partial assistance	9 (39.1%)	144 (38.3%)	1.45	0.50–4.21	0.548
Assistance	9 (39.1%)	114 (30.3%)	1.80	0.62–5.22	
Tube feeding	4 (17.4%)	39 (10.4%)	1.74	0.62–4.89	0.479
Hygiene					
Independent	5 (21.7%)	122 (%)			
Partial assistance	9 (39.1%)	140 (%)	1.53	0.53–4.46	0.51
Assistance	9 (39.1%)	114 (%)	1.86	0.64–5.39	
Toileting					
Independent	7 (30.4%)	162 (%)			
Partial assistance	7 (30.4%)	100 (%)	1.58	0.57–4.38	0.476
Assistance	9 (39.1%)	114 (%)	1.86	0.64–5.41	
Indwelling urinary catheter	8 (34.8%)	133 (%)	0.98	0.42–2.25	0.867
Above or diaper	3 (13.0%)	62 (%)	0.77	0.24–2.52	0.886
Oral care	13 (56.5%)	113 (%)	2.82	1.27–6.25	**<0.05**
Airway suction	7 (30.4%)	69 (%)	1.86	0.79–4.36	0.246
History of ICU admission	4 (17.4%)	68 (%)	0.96	0.34–2.73	0.845
Medical procedure					
Central venous catheter	3 (13.0%)	40 (10.6%)	1.24	0.38–4.01	0.988
Haemodialysis	5 (21.7%)	16 (4.3%)	5.00	2.06–12.15	**<0.01**
Surgical operation	5 (21.7%)	142 (37.8%)	0.48	0.18–1.26	0.186
Pressure ulcer	0 (0%)	18 (4.8%)	0.00	0–0	1.578
Endoscopic procedure	2 (8.7%)	32 (8.5%)	1.02	0.25–4.18	0.724
Drainages	2 (8.7%)	77 (20.5%)	0.39	0.09–1.61	0.268
Chemotherapy	1 (4.3%)	55 (14.6%)	0.28	0.04–2.02	0.285
Medical equipment					
Ventilator	1 (4.3%)	17 (4.5%)	0.96	0.14–6.75	0.632
Electrocardiogram	11 (47.8%)	122 (32.4%)	1.83	0.83–4.04	0.197
Antimicrobial exposure					
ABPC	9 (39.1%)	49 (13.0%)	3.78	1.72–8.33	3.78
ABPC/SBT	2 (8.7%)	24 (6.4%)	1.37	0.34–5.51	0.66
PIPC/TAZ	5 (21.7%)	15 (4.0%)	5.26	2.18–12.73	**<0.01**
CEZ	10 (43.5%)	104 (27.7%)	1.92	0.87–4.26	0.1
CTM or CMZ	2 (8.7%)	27 (7.2%)	1.22	0.30–4.93	0.78
CTRX or CTX	9 (39.1%)	111 (29.5%)	1.49	0.67–3.36	0.32
CFPM	3 (13.0%)	18 (4.8%)	2.70	0.87–8.37	0.08
MEPM	9 (39.1%)	30 (8.0%)	5.93	2.75–12.81	**<0.01**
LVFX or CPFX	1 (4.3%)	37 (9.8%)	0.43	0.06–3.12	0.38
AG	1 (4.3%)	11 (2.9%)	1.47	0.22–10.00	0.69
CLDM	3 (13.0%)	33 (8.8%)	1.51	0.47–4.85	0.48
GP	5 (21.7%)	19 (5.1%)	4.34	1.76–10.68	**<0.01**

Abbreviations: ABPC, ampicillin; ABPC/SBT, ampicillin/sulbactam; AG, aminoglycosides; CEZ, cefazolin; CFPM, cefepime; CLDM, clindamycin; CMZ, cefmetazole; CPFX, ciprofloxacin; CTM, cefotiam; CTRX, ceftriaxone; CTX, cefotaxime; 95%CI, 95% confidence interval; GP, glycopeptides; ICU, intensive care unit; LVFX, levofloxacin; MEPM, meropenem; PIPC/TAZ, piperacillin/tazobactam; RR, relative risk; VRE, vancomycin-resistant enterococci.

### Screening tests

Table 4 shows positivity rates for the five screening strategies implemented in each phase of the study period. Hospital-wide screening was performed a total of 32 times until Phase 3, with a positivity rate of 43/12,534 (0.34%). Admission screening was narrowed to high-risk individuals from Phase 3 onward, with a final positivity rate of 46/46,516 (0.10%). The positivity rates for antibiotic screening, passive screening, and dialysis patient screening were 31/9,230 (0.34%), 5/5,846 (0.09%), and 1/566 (0.18%), respectively. Hospital-wide screening and antibiotic screening had the highest detection rates. Of the 162 patients with positive results on screening, 46 (28.4%) were detected in admission screening, 43 (26.5%) in hospital-wide screening, and 31 (19.1%) in antimicrobial screening.

**Table 4.** Trends in new VRE detection rates by screening type for each of the four phases

Type of screening	Phase 1 May 2010 - Sep 2011	Phase 2 Oct 2011 - Aug 2013	Phase 3 Sep 2013 - Dec 2017	Phase 4 Jan 2018 - Mar 2021	Total
Hospital-wide screening	29/3186 (0.91%)	7/2675 (0.26%)	7/6673 (0.10%)	–	43/12534 (0.34%)
Admission screening	35/9355 (0.37%)	2/22194 (0.01%)	9/10687 (0.08%)	0/4280 (0.00%)	46/46516 (0.10%)
Antimicrobial screening	3/324 (0.93%)	11/1904 (0.58%)	17/3784 (0.45%)	0/3218 (0.00%)	31/9230 (0.34%)
Passive screening	0/410 (0.00%)	2/1300 (0.15%)	3/3525 (0.09%)	0/611 (0.00%)	5/5846 (0.09%)
Screening of haemodialysis patients	0/83 (0.00%)	1/417 (0.24%)	0/66 (0.00%)	–	1/566 (0.18%)
Other screening	33/698 (4.73%)	0/1258 (0.00%)	3/2056 (0.15%)	–	36/4012 (0.90%)
Total	100/14056 (0.71%)	23/29748 (0.08%)	39/26791 (0.15%)	0/8109 (0.00%)	162/78704 (0.21%)

Abbreviation: VRE, vancomycin-resistant enterococci.

### Phylogenetic tree analysis

**Figure 2.**
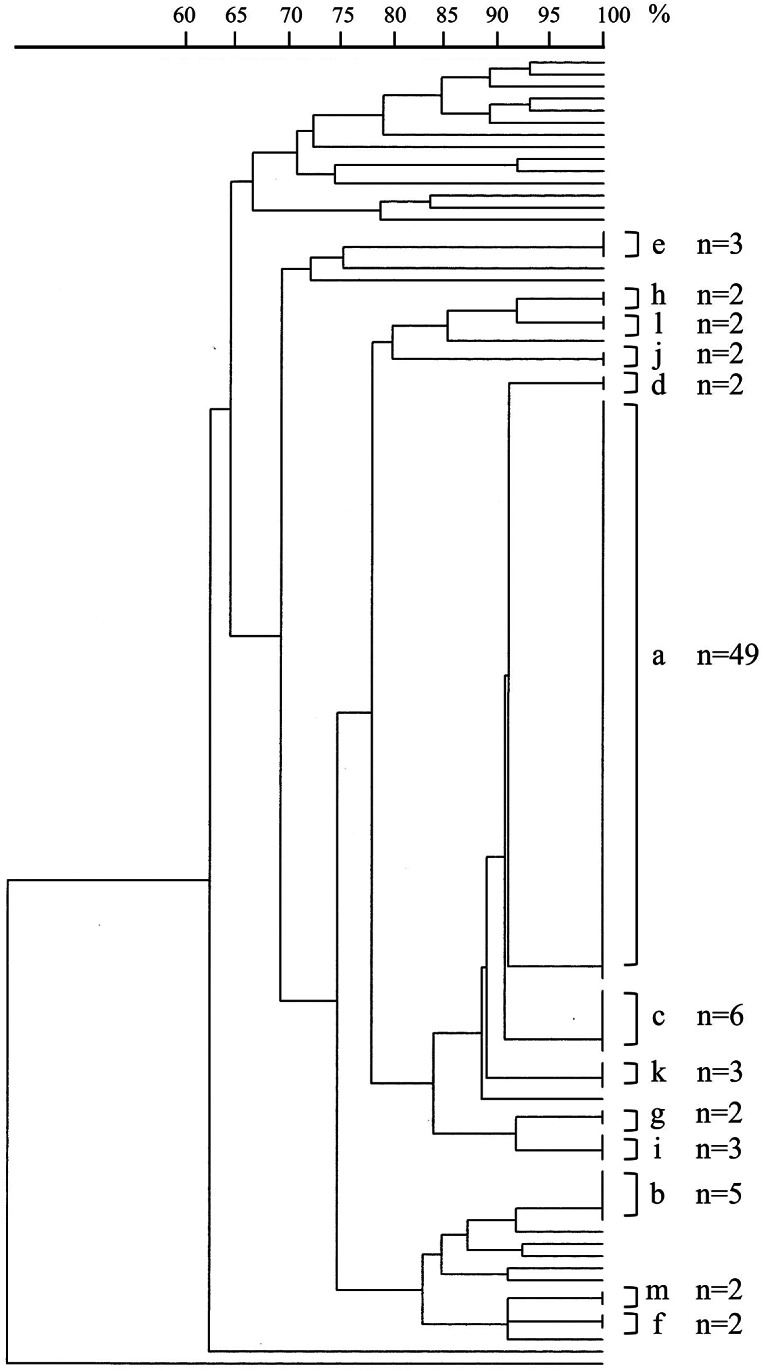
Molecular phylogenetic tree constructed from 109 vancomycin-resistant enterococci (106 clinical, three from toilet facilities) using pulsed-field gel electrophoresis (PFGE). Isolates with identical PFGE banding patterns were grouped and designated as groups a through m. Within group c (*n* = 6), three isolates originated from the toilet environment. In contrast, 26 isolates exhibited unique PFGE patterns, indicating that a total of 39 distinct PFGE types were identified across the isolates analysed.

To understand the relatedness of *Enterococcus* isolates, we performed PFGE and phylogenetic analysis on 106 VRE isolates from Phase 1 to 3 from the index case’s ward toilets (Figure 2). All isolates were identified as *Enterococcus faecium.* The results showed that 49 of 106 patient isolates (46.2%) shared the same PFGE pattern (group a), suggesting a high likelihood of horizontal transmission. Twelve additional minor groups (b through m) likely represented single strains. Notably, three group c isolates had a PFGE pattern identical to that of isolates from the toilet environment, indicating potential indirect transmission via the toilet environment. In contrast, 26 isolates (24.5%) displayed unique PFGE patterns. Overall, 39 distinct VRE lineages were identified during Phase 1. The *vanA* and *vanB* genes were detected in 96 isolates (90.6%) and nine isolates (8.5%), respectively, while one isolate could not be genotyped. All isolates belonging to clonal groups a through m, which represented multiple identical strains, carried the *vanA* gene.

## Discussion

We reported the characteristics of a large VRE outbreak involving over 100 cases, which was among the largest outbreaks reported in Japan at the time of its occurrence. VRE is recognized globally as a major healthcare threat, listed alongside methicillin-resistant *Staphylococcus aureus*, carbapenem-resistant Enterobacterales, and multidrug-resistant *Pseudomonas aeruginosa* by the United States (U.S.) Centers for Disease Control and Prevention [19]. In the U.S., VRE accounts for an estimated 5400 deaths annually and ranks among the leading pathogens responsible for healthcare-associated infections [20]. In Europe, surveillance data from the European Centre for Disease Prevention and Control indicate that the proportion of VRE has increased in several EU/EEA countries in recent years, although substantial inter-country variability persists [21, 22]. In 2010, resistance rates were 4.9% in China [23] and 24.9% in Taiwan [24]; recent data (2023) from the national Japan Nosocomial Infections Surveillance database showed a relatively low rate of 1.9% for *E. faecium* in Japan [25]. Despite this low prevalence, outbreaks such as that reported herein can place a large burden on healthcare systems and raise serious public health concerns.

Our retrospective case–control study, based on hospital-wide screening approximately 7 months after the index case, identified several risk factors for VRE colonization, including haemodialysis, oral care practices, and prior antimicrobial exposure. Consistent with U.S. studies [26, 27], we found strong associations between VRE carriage and exposure to vancomycin, carbapenems, glycopeptides, and piperacillin/tazobactam, likely owing to selective pressure and disruption of intestinal microbiota [28–31]. Patients with such antimicrobial histories consistently showed higher positivity rates (0.34%) across all outbreak phases, supporting their inclusion as a high-risk screening target.

Hospital-wide screening had a high positivity rate in Phase 1 (0.91%), which declined to 0.26% in Phase 2 and 0.10% in Phase 3, indicating reduced cost-effectiveness. Accordingly, this broad strategy was discontinued after two consecutive rounds without new VRE detections. Haemodialysis patients had a five-fold increased risk of VRE carriage, likely due to immunosuppression and frequent healthcare contact [32, 33]. Positivity remained low (0.18%) whilst intensive interventions were in place, including retraining in personal protective equipment use, enhanced disinfection, and biweekly screening. Nonetheless, the vulnerability of this population justified their continued inclusion in targeted screening.

Early detection of asymptomatic carriers is crucial because VRE can be acquired during hospitalization owing to antibiotic pressure and nosocomial transmission. Although admission screening is ideal, it has limitations; only 28.4% (46/162) of VRE-positive individuals in our study were identified at admission (Table 4). Most were detected through subsequent in-hospital screening. Given the limited sensitivity of a single rectal swab (56–70%) [34–36], repeated and multifaceted screening—particularly in high-risk patients – is essential to avoid missed carriers.

Screening methodology should also be considered when interpreting the declining positivity. Rectal swabs were cultured by direct plating onto selective agar, reflecting routine microbiological practice during the early outbreak period. Culture-based screening may be less sensitive than liquid-based or molecular methods, particularly for detecting low-density gastrointestinal colonization [37]. However, the same methodology was applied consistently throughout all outbreak phases, allowing internally consistent longitudinal comparisons. Moreover, the decline in screening positivity coincided with the disappearance of new clinical VRE infections and de-escalation of outbreak control measures, suggesting genuine epidemiological improvement rather than solely methodological under-detection. Several international frameworks, including guidance from the European Centre for Disease Prevention and Control, recommend risk-based screening and intensified surveillance during VRE outbreaks [38–40]. However, the optimal screening intensity and surveillance duration remain uncertain [41]. A meta-analysis suggested that cost-effectiveness depends on local variables such as prevalence, resource utilization, and infection control practices [42]. In our setting, new VRE cases emerged intermittently over 7 years, likely influenced by community reservoirs. Even a single case in low-prevalence countries like Japan warrants robust control measures. Given the potential for long-term intestinal carriage and ongoing community transmission, extended surveillance is advisable.

Unlike typical VRE outbreaks involving clonal expansion, we identified 39 genetically distinct strains via PFGE analysis of 106 isolates from infected patients. Although these strains may have originated from a common ancestor, subsequent genetic divergence likely contributed to the observed heterogeneity. Supporting this, a study at a healthcare facility in London reported the co-circulation of 45 distinct VRE strains within a single institution [43]. These findings should also be interpreted in light of the molecular typing method used in this study. Molecular typing was performed using PFGE; however, PFGE has lower discriminatory resolution than whole-genome sequencing (WGS) and cannot fully resolve fine-scale transmission events. Future studies using WGS may therefore provide more detailed insights into the genetic relatedness and transmission dynamics of VRE strains. Given that enterococci can asymptomatically colonize the gastrointestinal tract, it is plausible that multiple VRE strains were already present in the community before the outbreak was recognized. Supporting this possibility, a comparative analysis of antimicrobial-resistant bacteria in hospital versus municipal wastewater demonstrated higher concentrations of extended-spectrum β-lactamase-producing organisms and carbapenemase-producing *Enterobacteriaceae* in hospital effluent, whereas VRE levels were similar between hospital and municipal wastewater [44]. These findings suggest the potential for widespread VRE dissemination in the community. Although wastewater sampling was not performed in this study, previous wastewater surveillance studies suggest that VRE may circulate beyond healthcare settings [45]. *E. faecium* carrying *vanA* or *vanB* determinants has been detected in municipal wastewater and surface water, indicating environmental reservoirs of glycopeptide resistance. Genetic relatedness between wastewater and clinical isolates has also been reported [46], raising the possibility of bidirectional transmission between healthcare facilities and the community [47]. Consequently, a comprehensive strategy involving universal screening of all admitted patients and enhanced environmental cleaning and disinfection – rather than targeted screening and infection control alone – proved effective in mitigating VRE acquisition within our facility. Early implementation of broad-based screening during VRE outbreaks may therefore help identify previously unrecognized carriers.

The study period ended in early 2021 during the early phase of the COVID-19 pandemic. Recent surveillance reports suggest increasing VRE incidence in some regions in the post-pandemic period [48]. Changes in antimicrobial use, infection control practices, hospital occupancy, and patient movement during the pandemic may have influenced VRE epidemiology. Conversely, reductions in elective admissions and strengthened infection control measures may have temporarily reduced transmission opportunities. Therefore, the epidemiology observed in this study may not fully reflect post-pandemic trends.

This study has several limitations. First, due to the limited number of VRE-positive cases in our case–control study, we were unable to perform a multivariable analysis to adjust for potential confounders. Future research with larger sample sizes is needed to explore these relationships more thoroughly. Second, we relied on culture-based detection methods, which may be less sensitive than emerging molecular assays. Future studies should evaluate the cost-effectiveness of molecular techniques. Third, optimal screening strategies vary by region; our findings are most applicable to settings with low VRE prevalence and should be interpreted accordingly.

## Conclusion

Our findings demonstrate that no single screening strategy is universally optimal for VRE outbreak control. Instead, early implementation of broad-based screening is crucial to identify the true extent of transmission, particularly in low-prevalence settings where undetected colonization may sustain prolonged outbreaks. Subsequent dynamic, data-driven refinement of screening strategies based on positivity rates and local epidemiology enables more efficient allocation of infection control resources while maintaining effective containment. This adaptive approach provides a practical framework for the sustainable management of prolonged VRE outbreaks in healthcare settings.

## Data Availability

The data presented are available upon reasonable request from the corresponding author after publication.
